# Experiences of deliberate practice orientated psychological skills training for cancer care staff: Barriers and facilitators to learning and implementation in practice

**DOI:** 10.1177/13591053231210133

**Published:** 2023-11-14

**Authors:** Clement Boutry, James Rathbone, Felicity Gibbons, Dan Brooks, Nima Moghaddam, Chloe Mays, Priya Patel, Sam Malins

**Affiliations:** 1 University of Nottingham, UK; 2 Nottinghamshire Healthcare NHS Foundation Trust, UK; 3 Derbyshire Community Health Services NHS Foundation Trust, UK; 4 University of Lincoln, UK

**Keywords:** cancer, cancer care staff, deliberate practice, oncology, psychological therapy, training, wellbeing

## Abstract

This study explored participant-reported facilitators and barriers to learning and implementation from a 2-day training in psychological assessment and intervention skills for cancer staff, involving deliberate practice and supervision. Twenty-six semi-structured interviews were analysed using thematic analysis leading to four meta-themes: perceived practicality of training, impact of training: practice and its effects, implementation transfer processes and supervision engagement. Analysis identified a learning process to implementation: observation and practice of techniques during training facilitated participant learning; personal use and relevance of training content encouraged reflection, which enabled selection of appropriate tools for clinical practice; gains in commitment and confidence to use techniques supported participants to adapt clinical consultations, and supervision further facilitated implementation. Changing practice increased confidence, sense of achievement and engagement with participants’ own wellbeing. Interactive training, deliberate practice and continuous learning were facilitators to implementation whilst time constraint and low confidence in using techniques in remote consultations were barriers.

Compared to the general population, the prevalence of common mental health problems can be three times higher amongst people diagnosed with cancer (Linden et al., 2012), with a significant increase in suicide risk (Henson et al., 2019). However, this patient population is less likely to seek help for such problems, and signs can be missed by cancer care staff (Naylor et al., 2016). Untreated depression and anxiety can reduce cancer treatment effectiveness and patients’ quality of life whilst increasing healthcare costs (Melek and Norris, 2011; Mental Health Foundation, 2018). Therefore, clinical practice guidelines recommend training cancer care staff in psychological skills to assess mental health difficulties and deliver brief interventions (Merckaert et al., 2005; NICE, 2004).

Evidence on the effectiveness of such training and its impact on clinical practice remains underdeveloped, as is the current understanding of processes that translate such training into practice improvement. Whilst cancer care professionals attending training in psychological skills feel better prepared to provide emotional support (Delvaux et al., 2004) and more confident in identifying and managing psychological distress (Jenkins et al., 2010), existing research has focused on clinicians’ *experiences* of training and skills with less attention given to *implementation* into practice and contributing factors.

Ongoing supervision after training is deemed important to embedding learning of psychological skills in cancer care (Fixsen et al., 2006). The National Institute for Health and Care Excellence (NICE) recommends continuing support for professionals providing psychological care through supervision (NICE, 2004). A lack of post-training supervision may result in reduced skills and confidence to deliver them (Mannix et al., 2006). In oncology and palliative care, nurse specialists also reported supervision as a key training opportunity supporting them to practice taught skills and integrate them into routine care (Clark et al., 2015). However, current evidence on staff experience of supervision and training or the process through which they offer benefit is scarce. This is particularly important in the context of recently developed psychological skills training and supervision methods, such as deliberate practice, which involves identifying individualised skill learning needs and rehearsing them with feedback (Miller et al., 2023; Westra et al., 2021). This method can be effective and more time-efficient in psychological support skills training for cancer care staff, but a lack of process understanding means that reasons for such benefits remain unclear (Barrett-Naylor et al., 2020).

Staff who are more psychologically skilled may be at lower risk of burnout and have improved wellbeing (Morse et al., 2012; Ruiz and Odriozola-González, 2017). Nurses experience burnout at high rates, with cancer care arguably one of the most stressful working environments for healthcare professionals (Delvaux et al., 1988; Iacobucci, 2021; Peteet et al., 1989). Continuous exposure to work-related stress is a key risk factor for burnout, defined as workplace stress resulting from long-term involvement in emotionally-demanding situations causing fatigue and exhaustion (Kristensen et al., 2005). Given that nurses attending psychological skills training can experience less exhaustion and stress (Traeger et al., 2013) and may be more accepting of intervention outcomes (Delvaux et al., 2004), psychological skills training may contribute to burnout reduction. However, the mechanisms of such effects have not been explored (Hersch et al., 2016).

Overall, this study aimed to explore views of training participants with respect to (1) facilitators and barriers to implementation of psychological skills in practice, and (2) impact of training on clinicians’ practice and wellbeing.

## Method

### Design

A semi-structured interview design was applied from a critical realist epistemology, assuming that data were informative about, but not fully representative of, reality (Willig, 2019). Interviews focused on participants’ experiences of psychological skills training and how it impacted their practice.

### Training and supervision content and structure

Training was developed from the approach applied by Barrett-Naylor et al. (2020). This offered an initial training with equal focus on teaching assessment skills and brief intervention skills, primarily using deliberate practice to try the skills following demonstration and obtaining corrective feedback on identified individualised learning needs (Miller et al., 2023). A second training day was also offered 3 months after the initial day to update and consolidate learning, alongside optional monthly supervision sessions. Supervisions were 1-hour long for three-to-six participants and usually comprised of a self-compassion care space (Clark et al., 2022), a case discussion focused on a specific patient, and deliberate practice of identified skill learning needs (see Supplemental Material 1 for further information on training and supervision delivery and content).

### Participants

Eleven cohorts of cancer care staff were offered training and supervision in psychological assessment and intervention skills between November 2021 and July 2022. Overall, 145 cancer care staff working in the UK National Health Service (NHS) attended at least one psychological skills training day and were offered supervision sessions. Thirty-nine (26.9%) attended at least one supervision session and 26 (17.9%) consented to participate in interviews between June and August 2022. All interviewees attended both training sessions and 17 attended at least one supervision session; those who attended supervision attended a mean 2.5 sessions (Table 1).

**Table 1.** Participants’ demographics.

Participant role	*N* role	Average years in role (SD)
Cancer care coordinator	7	1.07 (0.89)
CNS	15	6.33 (7.53)
CNS Lead/Manager	2	1.25 (1.06)
Information/advice specialist	2	7.75 (10.25)
*N* total	26	4.63 (6.57)

CNS: Clinical Nurse Specialist; SD: standard deviation.

### Procedure

Training facilitators introduced the study to attendees directly prior to training. Those interested in participating gave consent to be contacted by an independent researcher (CB). Those giving consent to be contacted were subsequently emailed an information sheet and consent form. Participants had opportunity to ask questions before consenting to the 15-to-25-minute interview. Interviews were conducted by a researcher (CB) who was independent from the training delivery and participants’ work contexts. Interviews were audio-recorded and anonymously transcribed verbatim (see Supplemental Material 2 for interview topic guide).

### Method of analysis

Interview data were analysed using inductive thematic analysis, where themes were developed from the data following Braun and Clarke’s (2006) stages of analysis. The lead analyst (CB), who also conducted the interviews, began data familiarisation through reviewing the transcripts and keeping a reflective log. Initial codes were generated such that meaningful quotes were coded in a systematic way whilst updating the reflective log. In developing themes, related codes were grouped into meaningful categories, thereby creating potential themes, which were refined visually, using the reflective log to create a thematic ‘map’. Subthemes were broken down into comprehensive categories to define and name themes. To enhance quality assurance, a second analyst (CM), independently coded three transcripts and the themes identified were compared with those generated in the original analysis and differences resolved through discussion between coders to refine themes and ensure robust theme development processes (Nowell et al., 2017). A third analyst (PP), independent of the study, its conception and development, conducted an audit of the analysis and second coding. Differences were resolved by discussion and theme refinement. The analysis was organised using NVivo 12 software and themes relevant to the research question were reported with illustrative quotes.

This study used routine care service evaluation data. Ethical approval was not required, but the study was registered with the host NHS Trust.

## Results

**Figure 1.**
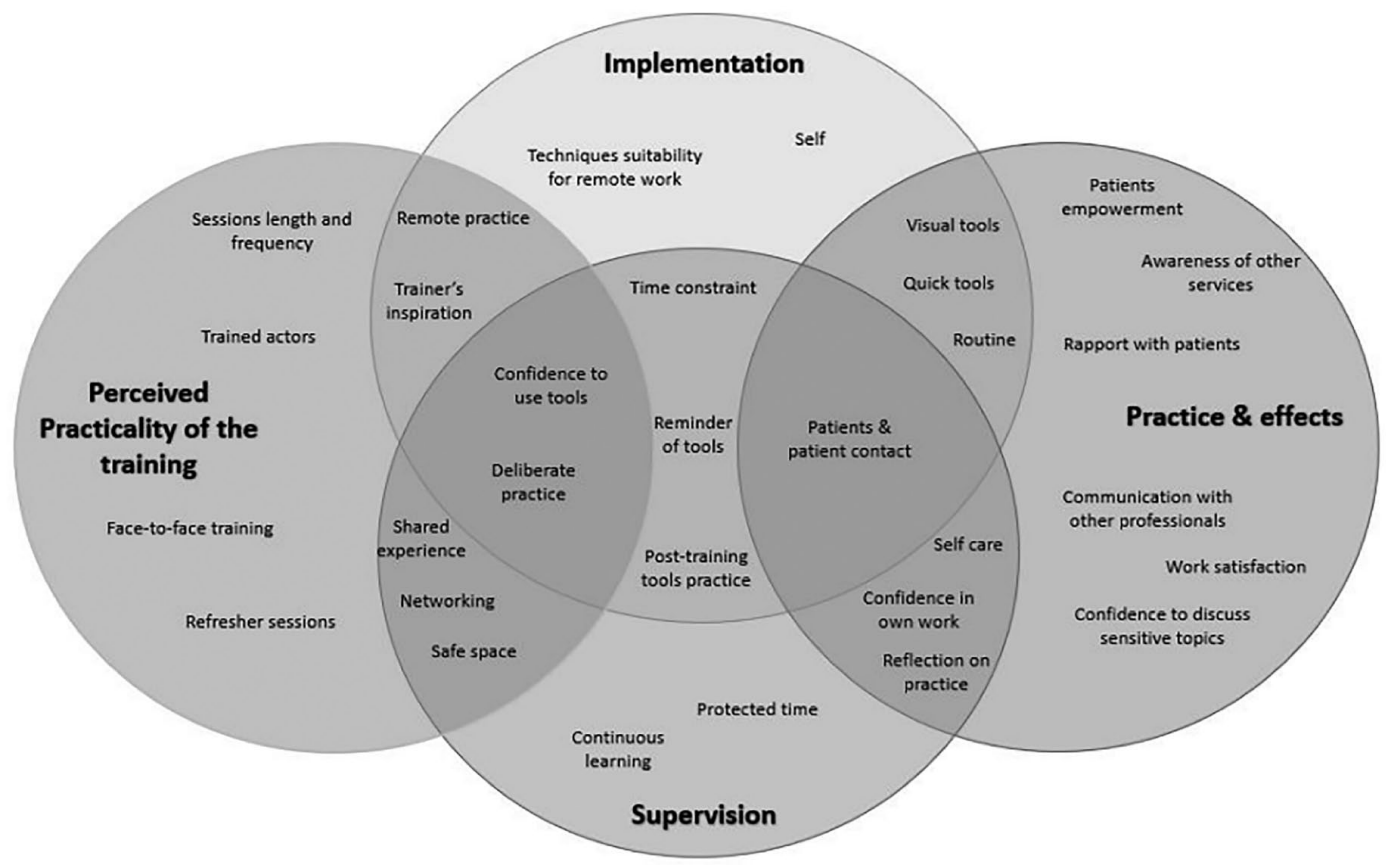
Ven diagram of themes.

Four interdependent, meta-themes relevant to the study were identified: perceived practicality of the training, impact of training: practice and its effects, implementation transfer processes and supervision engagement. They were distinctive but overlapping, mostly regarding the aspects of training and supervision that were key to implementation. Practicing techniques were identified in three of the themes (feedback and training quality, implementation and supervision). Similarly, characteristics of the self were reflected in all super-ordinate themes through sub-themes of: confidence in tool use, commitment to change practice and self-care (Figure 1).

The sections below report meta-themes (with a summary of meta-themes and themes, together with further quotes in Supplemental Material 3 and 4) and a learning process model.

### Meta-themes and themes

#### Perceived practicality of training

The way training was perceived played an important role for change in practice. Aspects of training reportedly facilitated implementation of skills, particularly the interactive focus, training content and training format. Other aspects did not, including lack of practice of remote consultations, low frequency of training updates and the need to change some tools for greater suitability.

#### Facilitators of learning outcomes

##### Interactive focus

The importance of interpersonal processes was highlighted, as the interactions in training sessions reportedly facilitated learning and increased the credibility of taught skills. The interactive approach encouraged networking with other clinicians and facilitated learning across professional roles and shared experiences. Specifically, ‘real play’ skills demonstration using trainers’ personal examples seemed to facilitate openness to learning amongst trainees, which may have established trust and a standard of sharing. Shaping and modelling sessions around openness to practice skills in small groups or pairs created ‘a space that you were not being judged for what you were saying’ such that participants ‘were made to feel comfortable asking questions’ (p011), discuss sensitive topics and practice skills which supported confidence and knowledge building. Adopting the attitude of using the training as a safe space to practice and rehearse skills without pressure to perform well alleviated some participants apprehension and initial discomfort with deliberate practice role play.

##### Training content and format

Participants described training content as practical and relevant to their roles, indicating that ‘it was helpful to [. . .] use examples of patients or even [. . .] your own life as well’ (p007). Training sessions were reported to be of adequate length, and frequency which, twinned with the structure allowed participants to learn the content, practice the skills during and after the sessions and feedback to enhance their learning experience. The face-to-face delivery of training permitted increased interactions. Rendering it a ‘really powerful’ (p002) necessary element that meant participants were ‘able to interact’ (p001).

#### Barriers to skills implementation

##### Remote practice

As several participants remained working predominantly with patients via telephone or video conferencing, some reported that using visual tools remotely was more difficult due to interpersonal processes such as explaining and describing them to patients who may ‘not be on the same page’ (p018). Whilst participants reported that work might be needed to adapt the tools for remote practice, they could ‘be just as helpful as being face-to-face’ (p016). Moreover, role plays could have been improved by incorporating remote consultation practice (i.e. not being able to see each other), such as simulating skills practice ‘over the phone, like ([. . .]) you have your backs turned to each other so you can’t see each other’ (p014). Participants also believed using trained actors playing patients would make practice more realistic and effective given that ‘practice with your colleagues is far less realistic’ (p004).

##### Tool and structure adaptations

Some participants reported that the volume of information delivered over 2 days was ‘a bit of an information overload’ (p006), which possibly prevented learning and skills adoption as there could be too much to remember and practice. Although some of the simpler, briefer tools were reported as unanimously helpful, usable and understandable (e.g. the stress bottle (Crawford et al., 2004)), participants gave mixed reports about more complex, time-consuming interventions such as the ADAPT problem-solving model (Attitude, Define, Alternative solutions, Predicting consequences, Trying out; Nezu et al., 2012). This was described as unclearly explained and, in some cases, not relevant to practice: ‘I think a lot of us in the room struggled to understand what information we were needing to pick out in the A and the D of ADAPT’ (p025).

##### Refresher sessions

Participants described wanting more ongoing training updates, once or twice a year, rather than seeing skills training as a discrete event, so that they could ‘refresh the skills that you learned during the course that you’ve just put to one side because you have got your comfort zone’ (p012). Routine refresher sessions were also perceived as an opportunity to expand their skillset if techniques ‘have been updated’ (p024) or with ‘any additions that we could take things a little bit further’ (p003).

### Impact of training: Practice and its effects

Participants reported that the positive effects of training on themselves personally and their practice was explained by changes in the way they related to their work, their patients and other professionals.

#### Patient relationships and dynamics

The most frequently reported impact of training related to patients. By actively listening and ‘empower[ing] patients to find their own solutions to problems’ (p026) and ‘get[ting] them to recognise the problems and what they realistically can do to help those problems’ (017), clinicians described improving their trusting rapport with patients: ‘It makes you think from the patient’s point of view about giving them the control’ (p013). As a result, they witnessed improved communication leading to better understanding of the situation, to which they responded in a ‘more supportive’ way (p016).

#### Self-confidence and perceived skill

Training increased awareness and knowledge of mental health: ‘it’s widened my knowledge a lot more on different tools that you can use and different ways about problem-solving and helping with stress management’ (p015). Participants also reported feeling better able to manage patients’ distress and sensitive topics: ‘[I now] ask questions that perhaps I would not have asked before like [. . .] suicidal risk’ (p005). Consequently, participants expressed ‘a better sense that I have actually done my job better’ (p011). Moreover, training instigated reflection on participants’ own practice and reflection ‘[about what] happened in the past that maybe I could have handled better’ (p002); whilst accepting that their interventions may not benefit every patient: ‘It is okay if [. . .] it was not as good as we hoped it would’ (p005). Furthermore, participants expressed gaining skills in managing boundaries in consultations and increased awareness of their own mental health as they used training content to manage personal challenges. More widely, participants reported that training positively affected their life outside of work such as ‘talk a bit more about how I’m feeling rather than bottling it all up’ (p016). Thus, training increased perceived skills and confidence in and out of work.

#### Other professionals

Participants applied the use of trained skills to the way they interacted with other professionals: ‘Just generally being more aware of my own wellbeing and the wellbeing of the people around me that are working alongside me’ (p025). This resulted in ‘building professional relationships’ (p002), particularly for those in leadership positions: By assuming more psychological care responsibilities, participants reported contributing to a reduction in referrals and waiting lists, or to be able to ‘help [patients] in some way even if [it is] while they’re waiting [to access a psychological care service]’ (p017).

### Implementation transfer processes

Interviews enquired about the facilitators and barriers to implementation. Sub-themes included: time constraints; practicing after training; participants’ commitment and confidence to use techniques; relevance to clinicians practice; transferability to individual clinicians’ practice (the type of patients that participants saw), and the support gained from training and supervision.

#### Time constraint

Time constraint was an organisational process recognised as the main barrier to implementation. Participants described not having capacity to explore the tools with patients due to other issues that needed to be covered, because some tools were deemed ‘quite time consuming, [. . .] especially the ADAPT’ (p003). Participants described having too many patients to use trained skills, partly because services were ‘not fully staffed’ (p021). At times, this caused negative emotions such as ‘get[ting] frustrated that I wish I had more time to spend with the patient’ (p014). Whilst having little control over time constraints, participants described the need to adapt their work and ‘change the service to improve the situation’ (p023). They aimed to routinely adopt tools, by seeing fewer patients for longer, and prioritising patients by needs. Beyond time with patients, participants also recognised that time to review training notes was salient for implementation ‘[to] have a little refresh of the stuff that we learnt’ (p015). However, time pressures and ‘being too busy’ (p016) prevented this process.

#### Practicing after training

Participants described that practicing skills in clinical and personal settings soon after training was a key factor for implementation through increased confidence to use the tools and ‘not floundering a little bit in there with a patient’ (p003). The rationale for practice was the transfer of confidence and skills to real-world settings: ‘It is about getting the confidence to use it, so it might be a case of trying this out on some friends and family, because you can use [trained skills] for any aspect in life’. (p008).

#### Commitment and confidence to use techniques

Participants believed that intrapersonal processes such as actively using the tools with patients and incorporating them into routine consultations was necessary: ‘It’s up to the [clinician] then how well they use those skills and the tools that they have been given’ (p009). This commitment was deemed necessary shortly after the training because ‘you are never going to get everything out of the training days’ (p014). Several factors were highlighted to motivate such effort. Firstly, participants’ experience of the benefits: ‘[I had] not realis[ed] how useful they could be and what other ways I could use the tool’ (p009). Personal preference and perceived relevance of different tools led to selection and implementation of some tools over others. Secondly, commitment to frequent use eventually increased confidence: ‘The more I use [the tools], the more confident I will become’ (p010). Conversely, fear that using the skills could ‘do more harm than good’ (p023) were barriers to implementations.

#### Format and relevance of the tools

The format and structure of tools and techniques taught were relevant to implementation likelihood. Consequently, training content had a direct impact on implementation as some tools were used over others: Typically, simple, visual tools were most readily implemented due to being easier to learn, understand and remember: ‘I personally like using pictorial aids because I think it helps patients, you can break things down with patients and that helps them see things when it is drawn out’ (p008). The stress bottle (a stress management intervention) illustrated these factors and was the most implemented tool by interviewees (*n* = 22): ‘[the stress bottle] just seemed like quite a nice simple and quick kind of way of doing it’ (p026). As most participants felt that the majority of the tools taught were appropriate to their role, tools that were not implemented were added as back up tools such that ‘[If] my go to tool wasn’t working I certainly would try another route, another tool’ (p012). However, one participant whose role was focused on assessing patients’ physical health commented that the tools taught were not relevant to their practice which was ‘very focused on how [patients] are coping with anticancer treatments’ (p021). Thus, training seemed more relevant where consideration mental health was part of the staff role.

#### Transfer to clinical practice

The nature of clinical practice and contact with patients was related to participants’ ability to implement skills and techniques into their practice. Specifically, not having a patient-facing role made opportunities ‘a little bit limited’ (p025) and some participants had ‘not come across a situation yet where I’ve had to use [the tools and techniques]’ (p015).

#### Training and supervision

Training and supervision also had an impact on implementation by motivating clinicians to reconsider their service structure in light of participants’ learning from the training: ‘I think it leads us to think how we need to run our service in the future, so that it can be more ideal for the patient and for us, to make us think that we are doing a better job’ (p023).

### Supervision engagement

Training attendees were encouraged to enrol in supervision, however only 27% took part. Interviews, therefore, explored the rationale for attendance (or otherwise). Time commitment prevented some participants from signing-up for supervision. Those who attended sought self-care and continued learning, which they achieved partly due to the safe space supervision was described as creating.

#### Rationale for supervision attendance

Time commitment was the main reason for not attending supervision, whether due to workload: ‘it’s just literally the workload that I’ve got at the moment that would get in the way’ (p004); childcare or other commitments such as ‘doing a module in my degree so that’s taken up one of my work days already’ (p021). Participants reported attending supervision primarily to reflect on their practice and continue ‘practising the techniques we’d learned and helping us actually put them into practice’ (p018). In addition, attendees expected emotional support, deemed ‘as important as looking after my patients because if I am not 100% on the game then I cannot give 100% to my patients’ (p026).

#### Supervision benefits

##### Course follow-up and practice

Supervision was viewed as a platform to continue to ‘familiarise ourselves with [training content]’ (p005) by discussing the training content whilst also being able to ask questions that arose after the training to ‘makes things a little bit clearer’ (p003). Supervision also offered a bitesize opportunity to continue ‘practising those little kind of tasks that we did within the training days’ (p015) with feedback and to consolidate knowledge. Supervision drew on interpersonal processes whilst being a forum to discuss participants’ patients ‘where perhaps things did not work so well or [. . .] what you could have done better’ (p005). Participants could reflect and get guidance on how to best support patients, whilst relating their situation to the training content. Furthermore, supervision attendees reported a sense of ‘reassurance that the tools that I’ve been using, I’ve been using them the correct way’ (p012) and increased confidence in their work with patients: ‘I felt more confident after the supervision and discussing it with live occasions that I have come across’ (p009). Feedback and learning from other attendees’ experiences was also reported as beneficial: ‘it helps getting different perspective from different people’ (p015).

##### Self-care

A crucial part of supervision was the focus on clinicians’ wellbeing. Attendees could discuss personal and work-related issues and reflect on how it affected them and their work. Given the nature of their roles working with cancer patients, offering a space to express their feelings was described as beneficial for their mental health: ‘I just feel a bit more supported, and a bit better equipped and a bit more mindful about the interactions that I have’ (p025); ‘it helped support us, which was something that we were crying out for, so that was really good. In practice, it can prevent burnout’ (p001). Before reflecting on their wellbeing during supervision, some attendees described being ‘so engrossed in your patient and their needs, [that] you forget the impact of what is happening to them and how that impacts on you as well’ (p012).

##### Safe space

Supervision created a ‘safe environment’ (p005), which was seen to empower participants to share difficult experiences and emotions. The safe space was defined as a place where participants could share feelings and were ‘not afraid to say if something is difficult or you have a difficult patient’ (p005).

### Learning process model

**Figure 2.**
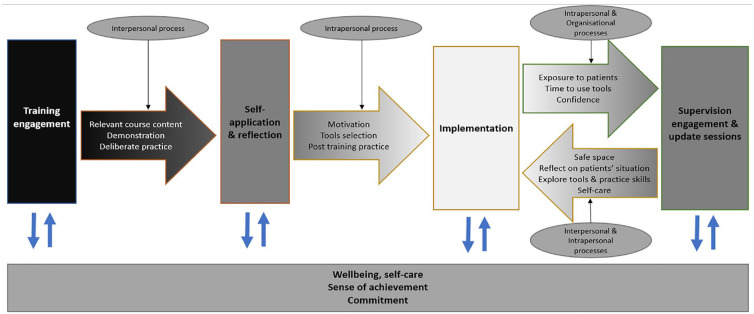
The learning and implementation process.

The themes described were developed into a learning process model that illustrated how participants reported the movement from training engagement to implementation and continuous practice improvement (Figure 2). This process incorporates intrapersonal (e.g. motivation), interpersonal (e.g. interaction with others) and organisational mechanisms (e.g. service set-up). Training engagement was facilitated by the personal and professional relevance of training content, and the opportunity to observe and practice skills. This in-turn facilitated post-training reflection, personal practice and use of skills that supported implementation to clinical practice. Use with patients helped practitioners grow in confidence with skills. Engagement in supervision and update training enabled practice improvement through reflective processes, supporting clinicians to tailor skills to patients’ situations, thereby creating a continuous learning and integration cycle. All processes were undergirded by participants relating the skills and their applicability to their own wellbeing. However, the different mechanisms of the process suggest that participants’ control over aspects of the implementation process vary.

## Discussion

This study aimed to understand facilitators to practice change following attendance at psychological skills training for cancer care staff, and changes in their own wellbeing. Participant accounts foreground common paths through which cancer care staff used training and supervision to acquire and implement psychological assessment and intervention skills, with specific characteristics of how training facilitated and obstructed learning and implementation. Participants highlighted the importance of an interactive training approach that demonstrated skills live and gave adequate opportunity to practice the skills with feedback. This approach improved engagement, confidence to try new skills and motivation for implementation. Skills’ relevance to participants’ own lives and a self-care focus helped validate their usefulness and general applicability, encouraging use of skills after training completion. Participants expressed that time limitations, especially for integration of longer techniques, and lack of adaptation to telephone use were barriers to implementation.

### Relationship to existing literature

#### Importance of deliberate practice

This study aligns with previous research highlighting the importance of deliberate practice and interactive, practical skill demonstration in effective, implementable training for psychological support skills (Barrett-Naylor et al., 2020; Rousmaniere et al., 2017; Westra et al., 2021). Specifically, attendees valued trainers’ demonstrations using real-life examples to facilitate understanding of tools and alleviate concerns regarding observed practice, whilst making the trainers more relatable. Consequently, participants reported being better able to personally conceptualise elements of the training content thereby increasing understanding. Participant reports also highlighted the importance of skills practice for ongoing implementation, which has been noted previously (Miller et al., 2004). Specifically, participants reported that beginning practice *during* training sessions increased the relevance, enjoyment and interaction within the group; and sometimes informally continued with friends, family and colleagues. The skill consolidation reported by those who attend post-training supervision also confirmed Miller et al.’s (2004) finding that post-training practice is of particular importance to implementation.

#### Self-application and self-reflection for implementation

The concept of the self was recurrent across themes. The importance of self-application and self-reflection for training implementation is replicated in previous research on self-confidence (Turner et al., 2011), implementation intentions (Gollwitzer, 1999) and self-efficacy (Machin and Fogarty, 2003): Practitioners’ intention to change their practice and confidence in their ability to apply training content affect the learning process leading to implementation; successful attempts at taught interventions enhanced confidence and resulted in further use. This study extends current findings by identifying differential factors that are relevant to the confidence in using training content from those impacting confidence in the techniques *themselves*. This was often based on personal experiences of techniques beyond work, which informed tool selection processes thereby introducing a key personal factor to post-training implementation.

#### Challenges related to dissemination of problem-solving therapy

Participant reports give an important nuance to dissemination of problem-solving therapy (Nezu et al., 2012). This is the best-evidenced psychological treatment delivered by non-therapists in cancer care (Sharpe et al., 2014; Strong et al., 2008; Walker et al., 2009) and is recommended as a key intervention component of psychological skills training for cancer care staff (NICE, 2004). However, some participants reported difficulty integrating this into routine consultations, considering it too complex for the time available, which was particularly pertinent as time-restrictions were the primary barrier to implementation. This suggests that without protected time for more comprehensive training and protected consultation time for delivery (as happened in the trials evidencing efficacy) problem-solving therapy may be challenging to implement in routine practice. This could explain why simpler, shorter tools were preferred in the context of the short training programme in this study and implementation into routine practice without additional consultation capacity.

#### Patients and care staff relationship

Using taught skills with patients, participants reported increased confidence in dealing with difficult situations and assessing mental health difficulties with improved patient rapport. These findings coincide with existing research demonstrating the increased confidence to identify and manage psychological distress through psychological skills training (Delvaux et al., 2004; Jenkins et al., 2010). This study further explored how such increased confidence was attained by practicing skills, the increased sense of achievement gained from work with patients and how increased confidence motivated further implementation.

#### Continued learning

Supervision was viewed as fundamental to the learning process amongst those who attended, acting as a hub that connected key practice change facilitators to enable continuous learning and enhance implementation. Beyond exploring and practicing tools, regular supervision created a learning cycle such that practitioners could continuously improve their skills by practicing and implementing more techniques over time. As such, supervision strengthened the training content, as previously described by Mannix et al. (2006). Therefore, skills training is a continuous process triggering change over time (Fixsen et al., 2006).

#### Caregiver wellbeing

This study may help explain the way training and supervision contribute to the reduction in the risk of burnout reported in existing literature, namely, through achieving greater work enjoyment, and confidence in practice outcomes (Duarte and Pinto-Gouveia, 2017; Ross et al., 2017). Participants reported that training was salient in improving their own wellbeing because it increased mental health awareness and introduced techniques with potential to benefit participants personally. Supervision also instigated self-reflection, particularly regarding the work-environment’s impact on participants personally, and offered a perceived safe place to share experiences.

### Limitations

Supervision was highlighted as key to enhancing implementation by some participants. Yet several interviewees did not attend supervision. Therefore, themes relating to supervision may not be applicable to the majority of training attendees.

### Clinical implications

Psychological skills training in cancer care can benefit from live demonstration of brief, simple, visual skills that can be adapted to remote consultations. Training should include time for attendees to practice taught skills and receive feedback. Attention should be given to implementation as early as possible, encouraging practice soon after training and attendance at supervision. Training could make explicit reference to the potential for personal benefit from attendance and encourage application of skills to both work and non-work situations, due to potential for general benefits. Adaptation of techniques should be encouraged for the wide range of settings and roles in cancer care where psychological skills may be relevant. Overall, participants can be encouraged that the time commitment to learn and apply psychological skills may enhance both their personal and professional lives.

### Future research

This study investigated the combined experience of training and supervision, although interview data suggest that each may have distinct contributions to implementation. Future research could explore the unique contributions of each practice to support improved supervision and training design.

Although the interview topic guide did not explicitly ask about burnout or wellbeing, these emerged as important themes. Participants reflected on factors contributing to stress reduction, improved wellbeing and work satisfaction. This indicates that psychological skills training could provide the double-benefit of upskilling the cancer care workforce and improving workforce resilience. Future work focused on the possible contributions of skills training to resilience and coping could enable a clearer understanding of potential beneficence and how this might be harnessed.

## Conclusion

This study presents the processes of learning and implementing psychological assessment and intervention skills in cancer care following brief training and supervision, based on participants accounts. It suggests that interactive training, deliberate practice and continuous learning processes such as supervision, facilitate learning and implementation by supporting greater confidence and motivation to try taught skills after training completion, even in participants’ personal lives. Furthermore, this study showed the importance of practitioners’ characteristics such as confidence, motivation and commitment for change, as contributors to implementation.

## Supplemental Material

sj-docx-1-hpq-10.1177_13591053231210133 – Supplemental material for Experiences of deliberate practice orientated psychological skills training for cancer care staff: Barriers and facilitators to learning and implementation in practice Supplemental material, sj-docx-1-hpq-10.1177_13591053231210133 for Experiences of deliberate practice orientated psychological skills training for cancer care staff: Barriers and facilitators to learning and implementation in practice by Clement Boutry, James Rathbone, Felicity Gibbons, Dan Brooks, Nima Moghaddam, Chloe Mays, Priya Patel and Sam Malins in Journal of Health Psychology
