# Supplementary material for: Experiences of deliberate practice orientated psychological skills training for cancer care staff: Barriers and facilitators to learning and implementation in practice
Source: J Health Psychol. 2023 Nov 14;29(5):467–80. doi: 10.1177/13591053231210133 (PMC11005300; doi:10.1177/13591053231210133)
Supplement: sj-docx-1-hpq-10.1177_13591053231210133 – Supplemental material for Experiences of deliberate practice orientated psychological skills training for cancer care staff: Barriers and facilitators to learning and implementation in practice [file sj-docx-1-hpq-10.1177_13591053231210133.docx]

**Experiences of Psychological Skills Training for Cancer Care Staff: Barriers and Facilitators to Learning and Implementation in Practice**

# Supplementary Material

1: *Training and supervision*

Eleven cohorts of cancer care staff were offered training and supervision in psychological assessment and intervention skills between November 2021 and July 2022. This included 145 participants who attended at least one of the two training days offered and 28 (19.7%) participants attended at least one follow-up supervision session.

The training focused on “real-play” demonstration of the skills and techniques by the facilitators followed by training attendees skills practice and refinement using corrective feedback from attendees with an observational role and facilitators. The training focused on skills and techniques that could be delivered in between 5 and 15 minutes due to consistent feedback about lack of time in practice being a barrier to implementation.

The techniques were adapted from therapeutic models such as Cognitive Behavioral Therapy and solution-focused brief therapy (De Shazer & Coulter, 2012), Acceptance and Commitment Therapy (Hayes et al., 2012)with values-based action identification and goal setting, Problem-Solving Therapy (Nezu et al., 2012) with the ADAPT model and Motivational Interviewing (Miller & Rollnick, 2012) to improve overall active listening skills and facilitate change.

Following attendance to the first session, participants were offered monthly, one-hour group supervision remotely via video. Supervision comprised three components, each aiming to be approximately 20 minutes in length: a self-compassion care space (Clark et al., 2021); discussion of a specific patient and associated issues, then deliberate practice of identified skills deficits.

## 2*: Interview topic guide*

|  | Question | Probes |
| --- | --- | --- |
| 1 | How did you hear about the training programme? What motivated you to take part? | Searched for it / Line Manager / CPD Decision process Considered before Personal interest Need for training |
| 2 | What did you think of the training sessions? What was helpful / unhelpful? What was usable / relevant to your practice? | Likes / dislikes Expectations Trio practice Variety of tools (any preference?) Practicable / action-oriented Experience Length of sessions Frequency of sessions Being involved in a research project |
| 3 | What impact has the training had on your practice?  What tools or skills taught in training have you managed to apply to your practice? Is there any area of the training that did not affect your work? What negative impacts did the training have on your work? | How did the training make a difference on practice? Which tools were used? How often? How soon after training? Reasons for using them or not? Were they useful? Why? Achievement How did it feel? Confidence? Practice with others? Benefit for patient Benefit for practice Benefit in own work (e.g., work engagement / enjoyment) If not used in practice or no effect: reasons for it |
| 4 | What are helped you to use the skills and tools taught in training? What got in the way of you using the tools and skills taught in training? What could support the implementation of those skills into your practice? | Relevance Practice Support from trainers Follow up sessions Supervision What has helped you using those skills What has not helped using those skills What support have your received |
| 5 | Did you take part in the supervision programme?  What motivated your decision? If not attended: What could have changed your mind? | What did you expect from it? Expectation met? Have you benefited from it? How? Likes / dislikes How did it fit with training? How did it fit with practice? If not signed up: Any support/information we could have provided? |
| 6 | Is there anything that you would change in the training programme? | Training sessions  Supervisions  Number of sessions  Structure of the sessions  Practice  Need for a refresher  Implementation into practice |

3: *Themes and Meta-themes*

| Meta-themes | Themes |
| --- | --- |
| Perceived practicality of the training | Facilitators of learning outcomes  Training barriers to skills implementation |
| Impact of the training practice and its effects | Patient relationships and dynamics  Self confidence and perceived skills  Other professionals |
| Implementation transfer processes | Time constraint  Practicing after training  Commitment and confidence to use the techniques  Format and relevance  Transferring the tools to clinical practice  Training and supervision |

## *4:Further quotes*

| Theme and sub-theme | | Illustrative quote |
| --- | --- | --- |
| Feedback and training quality |  | |
| What Worked Well - Interactive Focus | [The trainers] were very personable people and that came across and I think if you can like the people that's leading the sessions it certainly helps you maintain interest because these things are not easy and it takes a lot of thinking, a lot of concentration especially when it can be a bit out of your comfort zone, […] You can see how good it can be, especially with practice, and it was at times quite inspiring what you could, how they led the training. (Participant 24) | |
|  | You didn't feel judged, it felt like everybody was in the same place and again it wasn't... You weren't being put on the spot, you weren't having to play it out in front of the whole group, you were doing it in your own group and I think that to me was very helpful, it didn't feel like you were exposed, it felt like it was almost a safe space to practice. (Participant 2) | |
| What Worked Well - Training Content and Format | I enjoyed the fact that it was practical, that we did come away with ideas and helpful hints on how we can then talk to our patients about [improving their concerns], they were very powerful. (Participant 20) | |
|  | I thought the facilitators targeted it at the groups that we were for Prehab really well so they kept referring back to how we would use these skills with our group of patients and they are quite unique or they are preparing for cancer treatment but they might feel very overwhelmed with the new diagnosis and also with the motivation side of things to get them to engage with us and both days of the training were targeted really, really well to us. (Participant 14) | |
| Improvement Suggestions - Remote Practice | Sometimes it's difficult on the phone, you don't know whether it's gone quiet because there's a problem with the line […], or it's actually because they're upset (Participant 17) | |
|  | I suppose we could have done exercises of being over the phone and not seeing the person next to us, maybe a shield or just having a go at that to see how it would implement and how we could change it. (Participant 6) | |
|  | Sometimes it's hard to practice the psychology skills with somebody that you work with and that you know well, it's hard to kind of switch off and oh I know this is fake, whereas when it's with an actor, you very, very quickly feel that oh this is a real situation. (Participant 14) | |
|  | WOOP [a goal setting intervention] […] I find that's much easier to do over the phone and to be on the same page with someone for there to be no misunderstandings about where someone's at or what their goal is. (Participant 18) | |
| Impact of Training: Practice and its Effects |  | |
| Patients Relationships and Dynamics | As a [clinician] by background and you have got that problem solving nature, you want to just go in and fix things and [the training has] given you a way to actually structure it and help the patient come up with solutions of their own or help them feel that they have got a bit more control back. (Participant 14) | |
|  | Instead of it being intense, “I'm having a chat with a professional”, it's made them feel more comfortable doing it like this and maybe it wasn't as like doctor/patient view, it was more of a, “oh they are here to help me” so they've felt able to open up more. (Participant 22) | |
| Self-confidence and Perceived Skill | It helps me feel more comfortable to actually feel like I made a difference for a patient and to have actually given them a tangible realistic way to go about solving a problem they might be having, so I think it's just made me more confident in helping patients (Participant 18) | |
|  | One of the things that did really help me is letting me know that it is okay to put a time constraint on a conversation. […] That it does sort of direct the call and that conversation a lot better if you can sort of say we have only got so long to talk. (Participant 11) | |
| Other Professionals | I'm doing a lot of communicating with clinicians and professionals and that actually I've still been able to apply some of the training to actually those meetings, reflecting on how I communicate with professionals as well as just patients, and actually I've been able apply some of the communication and techniques actually into a clinical setting as well, into professional meetings. (Participant 2) | |
|  | I would be able to help them and support them a little bit more whereas previously I would probably have just literally, oh I'm really sorry, I'll get the nurse to give you a call back because I can't help. (Participant 16) | |
|  | Without me having to go to the nurse all the time, I'm able to I say deal with it, it's a bit of a strong word, but I can manage it a little bit better myself so the nurse can obviously prioritise other patients that need it. (Participant 16) | |
| Implementation |  | |
| Time Constraint | It's having the time with the patients to be able to obviously sit down and actually do it all. It's just sort of time constraints. It's not that the things take too long, it's just as in the actual time that other professionals have with the patient. (Participant 22) | |
|  | It is just that we are so short staffed, you do sometimes feel like we are rushing everything now whatever we are doing now. (Participant 13) | |
| Motivation and Commitment | “If you do not start to use them then quite quickly then you do not use them.” (Participant 11); | |
|  | [If you do not practice the tools shortly after training,] you won't remember everything, and you are never going to get everything out of the training days. (Participant 14) | |
|  | So in a way from that I felt more confident with the knowledge that I'd gained, to use it, but actually putting it in practice, I was a bit nervous. I wanted it to be a seamless transaction for the patient. Not a transaction, but I didn't want them to know that I was a bit nervous. (Participant 1) | |
| Format and Relevance of the Tools | I think just having that visual with the patient is really, helps them to understand what you are trying to say and that you are not trying to just say oh if we take out a bit out it will be better. (Participant 14) | |
|  | They are very visual and a lot of my patients prefer to see what is causing their stress and anxiety, and what they can and cannot control rather than just talking about it. (Participant 27) | |
|  | It is good to be learning about those different tools because you know if you have got a really difficult patient or a patient with lots of issues, it just opens up that opportunity to try something if you have tried everything else, it just gives you more tools in the arsenal really. (Participant 8) | |
|  | I have been a clinical nurse specialist for a long time but my current role is very focused, my clinical work with patients is very focused on how they are coping with anticancer treatments that they receive and I think anxiety/depression issues may come out in conversation but my role is very different to a lot of the other traditional nurse specialist roles that have a holistic view with patients and their conversations will be about the grit or impact I think of their diagnosis on their lifestyle and that holistic review and I think perhaps that leans more towards using the stress bottle as assessment whereas I have tended to fall into using the anxiety and depression scores and making referrals. (Participant 21) | |
| Transfer to Clinical Practice | My time unfortunately has been doing other things like service reconstruction and things so I have not had that same time with the patient that would give me the chance to do those things. […] I haven't really spoken to many patients so I think that's the problem where that lies. (Participant 24) | |
| Training and Supervision | “Seeing it in practice when they were showing us, [we were] able to see the difference very easily, and how we felt, and then to put that in line with how our patient would feel” (Participant 1) | |
|  | I think it was just like we said the energy from the people that were actually doing the training, that this actually works and it does work so you know yes we can use it and yes it can have a massive impact on somebody's life, so let's go out, let's try it, let's see how that works. (Participant 20) | |
|  | Training itself was quite motivating so it did make you want to go out and you could sort of see the benefit that it would have for your patients, so it [was] quite inspiring to want to go out and try and do some of it. (Participant 4) | |
| Supervision Engagement |  | |
| Rationale for Supervision Attendance | When it's been taken place our staffing is terrible at the minute, so we've been, we've not had the capacity for me to be able to have that time if that makes sense.[…] When it's been taken place our staffing is terrible at the minute, so we've been, we've not had the capacity for me to be able to have that time if that makes sense. (Participant 7) | |
|  | Over recent months I have also been studying and had quite a lot on really so I think it was just a additional time pressure I think on this occasion but I know it is being looked at for our job plans and I am hopeful that this will come to fruition in the longer term, I just haven't had time recently. (Participant 21) | |
|  | Just with annual leave, obviously it's school holidays, so it's just very strapped. […] If it hadn't been the school holidays it might have been slightly different and obviously we've got a holiday booked as well, so it's just sort of the timing's not very great over the school holidays. (Participant 22) | |
|  | [to talk] about things and about situations you have been in because sometimes I find talking about something is a better way of reflecting. I find reflecting my practice is better to talk to somebody and have that interaction rather than just doing a written reflection. (Participant 11) | |
|  | I was very eager to not only support myself but also support my colleagues because you know you recognise how important that is for your own mental health and your own wellbeing, but again it proved when we were doing it as a peer to peer group support, it proved very difficult for us to actually leave our work commitments behind and actually place that much importance on those supervision sessions and even I'm guilty of that, saying well actually I'm too busy, I can't do this right now, but I think if it's planned within our work commitments and planned as an hour at least once a month to go and have that supervision, you should actually have that planned within your workload because without it, yeah you do tend to find that you do get overwhelmed with emotions sometimes and you know you do have to put in your own barriers within your practice to be able to practice safely and I think that supervision will all be able to help us practice safely and effectively. (Participant 20) | |
| Supervision Benefits | I really found it quite helpful having the sessions broken down into smaller groups over teams just to keep an eye on us and then it was the re-discussing things that we had done and then it was "oh, I forgot about that one", so it brought it back to the forefront of your memory. […] The supervision really helped me because there were things that maybe I had forgotten with it being such an informationally packed two days. (Participant 6) | |
|  | Yes and then when you have had your supervision meetings and you talked about case studies of the tools you have used and how you would use them and the outcome of that and it was just nice to get the clarification that actually yeah, we can see that you use that tool and we can see that it worked. (Participant 12) | |
|  | It was put into to help support us with clients and with ourselves as well, so it was just part of our job role to be able to have a space where we can talk about clients and have some feedback on that as well. (Participant 10) | |
|  | It's just that extension of that, it's just the extension of the course really, it's not just a one hit and that's it, you're done, it's nice to have that input afterwards to be able to ask questions that may come to mind after you've attended the sessions. (Participant 26) | |
|  | I kind of used it and I would say probably a 6 out of 10 understood what I was meant to be doing and then after the supervision I got a better idea of that there is a way of using it and what other things I could add. (Participant 9) | |
|  | I would say that getting relevant training sessions and the supervision just kind of continues that on and knowing that you can, if you have a difficult situation, if you tried one of the skills out and it had gone not as planned, that you would have that time that you can bring it up and get some guidance on it was really good. (Participant 14) | |
|  | It just gives you that opportunity to again say something didn't go quite right, how did somebody else deal with it, or what would somebody else do in their situation, so again it's reiterating that things aren't, you know, text book perfect, life isn't, so again it's giving people that reassurance it's okay to learn. (Participant 2) | |
|  | That scenario popped up for me I didn't know how to be able to support someone who appeared clinically very distressed but wouldn't talk to me and usually you would need quite a long time to be able to spend with that person to be able to help them properly so at that point I was given the bull's eye [a value clarification exercise] process with all the information down and she said that it would be okay, to leave, as long as I explained it properly then I could leave that information with the patient. (Participant 9) | |
|  | I think if there is any uncertainty about anything, if you have got a question, if you are stuck, if you sort of feel as though you have hit a brick wall and think "right, which way do I go next, I am not quite sure with this, I am not getting there", having that backup there is useful. (Participant 3) | |
|  | We would basically get together to talk about our experiences so far and what we have used and what we found helpful and then it was being able to feel confident enough to practice those ourselves to our own patients. [other clinicians] were like "I had this episode happen last week and this is how I dealt with it", and we all found that really helpful because "oh yes, I forgot about that one" and it was something that we can take into our practice as well. (Participant 6) | |
|  | I don't deliver as many sessions as the other people that I work with, with me being a manager whereas the others are delivery staff, so it was good to be able to listen to them and how they've dealt with situations as well. (Participant 10) | |
|  | It just gives you a chance to listen to each other and think about what can I do to look after my own wellbeing. (Participant 14) | |
|  | It just keeps me in check with my own wellbeing. (Participant 12) | |
|  | If you going through a difficult time at work or at home then, there was something one day but I cant remember what it was, I think it was just the pressures of work but it was just discussing that and visualising things, what you were going to do at the weekend and things like that, just to ground yourself again really. (Participant 13) | |
|  | It stops you worrying about things too much like taking stuff home, I mean you still do think about things but I think when you're able to discuss it in that kind of setting, it just helps you to allow your emotions to be felt. (Participant 23) | |
|  | It has been nice to talk to [trainer/supervisor] and also my colleagues so I understand that I am not the only one that may have these challenges. (Participant 27) | |
|  | I personally did not have an outlet for what I was expressing, and I felt very conflicted with the fact that I have never said anything and never stood up for something when my values were that I would always speak up.  And that was affecting me because I felt like I was not true to myself and it was affecting me. All of this I was not aware of.  Being able to speak to somebody in the supervision, and being able to be guided and supported into making this realisation, it made me feel like a weight had been lifted off my shoulders. (Participant 1) | |
|  | It gives you the safe space to talk about situations and it is a safe space, and it's a safe space to say, this happened, you know, what could I have done better, or how could I have managed this, and it gives you that opportunity to talk through within all of you, your past experiences, how things have gone well, not well before. (Participant 2) | |
|  | It was put into to help support us with clients and with ourselves as well, so it was just part of our job role to be able to have a space where we can talk about clients and have some feedback on that as well, and kind of open up. (Participant 10) | |
|  | That blocked out supervision time facilitated by someone who is not on the ground with us, understands the service, knows what is happening but doesn't know the day-to-day things and doesn't need to know the day-to-day things so that was really helpful having a, I don't want to call them an outsider because he wasn't an outsider, but if that makes sense that someone.  (Participant 14) | |
|  | Added the quote we discussed, but did not mention it in the text as it is a single quote. It goes with "not being afraid to express themselves" and is therefore somewhat reflected in the text. | |

## References

Clark, M., Bradley, A., Simms, L., Waites, B., Scott, A., Jones, C., Dodd, P., Howell, T., & Tinsley, G. (2021). Cultivating compassion through compassion circles: Learning from experience in mental health care in the NHS. *The Journal of Mental Health Training, Education and Practice, 17*(1), 73-86. <https://doi.org/10.1108/JMHTEP-03-2021-0030>

De Shazer, S., & Coulter, M. (2012). *More than miracles: The state of the art of solution-focused brief therapy*. Routledge.

Hayes, S. C., Strosahl, K., & Wilson, K. G. (2012). *Acceptance and Commitment Therapy: The Process and Practice of Mindful Change* (2nd ed.). The Guilford Press.

Iacobucci, G. (2021). Staff burnout: MPs demand “total overhaul” of NHS workforce planning. *BMJ, 373*(n1461). <https://doi.org//10.1136/bmj.n1461>

Miller, W. R., & Rollnick, S. (2012). *Motivational interviewing: Helping people change*. Guilford press.

Nezu, A. M., Nezu, C. M., & D'Zurilla, T. (2012). *Problem-solving therapy: A treatment manual*. Springer.
